# Application of Osteopathic Manipulative Treatment (OMT) in Neurodegenerative Disorders: A Scoping Review

**DOI:** 10.7759/cureus.94748

**Published:** 2025-10-16

**Authors:** Julia P Bethea, Hasin Sharma, Nicholas Doberstein, Tara Shenker, Bradley Gregory, Rebecca Hoffman, Daniel Aizenman, George Guirguis, Johnny Hoffmann, Snober Tazani, Zachary Harris, Joshua Costin

**Affiliations:** 1 Department of Medicine, Dr. Kiran C. Patel College of Osteopathic Medicine, Nova Southeastern University, Clearwater, USA; 2 Department of Medical Education, Nova Southeastern University Dr. Kiran C. Patel College of Allopathic Medicine, Fort Lauderdale, USA

**Keywords:** alzheimer's dementia, alzheimer's disease, amyotrophic lateral sclerosis, huntington’s disease, neurodegenerative disorders, osteopathic manipulative medicine (omm), osteopathic manipulative therapy, parkinson' s disease, neurology

## Abstract

Neurodegenerative diseases are comprised of a host of chronic conditions that impair the central nervous system. Osteopathic manipulative treatment (OMT) consists of many non-invasive modalities that can be used to treat a wide variety of ailments and conditions. OMT is reported to increase the range of motion and lymphatic flow, as well as decrease pain in a wide array of disorders. However, the efficacy of using OMT in neurodegenerative disorders has not been well established.

The objective of this scoping review is to map the evidence that pertains to the application of OMT in treating neurodegenerative disorders and identify the gaps in the literature on this subject. This study was designed according to the Joanna Briggs Institute (JBI) guidelines for scoping reviews to gather information on OMT’s potential efficacy in managing Parkinson’s disease (PD), Alzheimer’s disease (AD) dementia, amyotrophic lateral sclerosis (ALS), and Huntington’s disease (HD). Peer-reviewed literature was collected through the Excerpta Medica database (EMBASE), Ovid Medical Literature Analysis and Retrieval System Online (MEDLINE), and Web of Science. The criteria were limited to papers in English published between 1999 and 2023. The following search string was utilized: “osteopathic manipulative treatment” OR “osteopathic manipulation” AND “neurodegenerative disorders” OR “Alzheimer’s disease” OR "dementia” OR “amyotrophic lateral sclerosis” OR “Parkinson’s disease” OR “Huntington’s chorea”.

One hundred and forty-three articles were identified following final screening and critical appraisal, with eleven articles selected for analysis in this study. Data from the selected articles demonstrated that OMT can possibly attenuate symptoms in patients diagnosed with neurodegenerative diseases. Studies in rats showed that OMT techniques were found to alter cholinergic neuronal genes, improve spatial learning and memory, reduce amyloid β protein levels, modulate synaptic transmission, and increase levels of the cytokines IL-1, IL-10, IL-13, RANTES, IL-17A, and EOTAXIN effects in AD dementia. ALS patients demonstrated a high level of satisfaction with OMT and did not report any adverse effects, though there was no decrease in pain or increased quality of life reported. PD patients reported improved postural stability, balance, and gait after OMT. No results were returned regarding OMT’s effects on HD.

Preliminary results in human PD and ALS patients who received OMT as an adjunct to traditional treatment regimens showed promising results, though few studies were found that address the topic, and the sample sizes of the studies that were found were small. There were no studies of the effects of OMT on human patients with AD or HD found, though preclinical studies in rats supported their trial in subsequent human studies. While current research on the impact of OMT on these neurodegenerative diseases is promising, there remain large gaps in the literature. Further research is necessary to support the use of and long-term efficacy of OMT in neurodegenerative diseases.

## Introduction and background

Neurodegenerative diseases are a growing concern and have contributed to globally increased mortality rates in recent years [1]. Alzheimer’s disease (AD) and other dementias were ranked seventh in the global leading causes of death in 2021 [1]. In high-income countries, they are ranked as the fourth leading cause of death [1]. Neurodegenerative diseases are defined by damage to, and consequent loss of, neurons through the aging process as well as genetic and environmental factors [2]. This progressive damage is what causes pathological abnormalities found in various neurodegenerative diseases such as Parkinson’s disease (PD), AD dementia, amyotrophic lateral sclerosis (ALS), and Huntington’s disease (HD) [2].

Of these diseases, AD and PD are the most common. Given that the most significant risk factor for neurodegeneration is age, AD and PD most often occur in the elderly population. The United States population over the age of 65 years old is estimated to increase from 53 million people in 2018 to over 88 million in 2050. The aging population bolsters the need to improve current treatment and care plans for age-related health conditions [3]. 

PD is expected to grow to more than one million cases in the United States by 2030 [4]. The pathophysiology of PD is characterized by an overall decreased dopamine concentration in the substantia nigra, resulting in the classical motor symptoms of the disease, including bradykinesia, rigidity, rest tremors, or postural instability [5]. The presence of microscopic Lewy bodies in neuronal cell bodies specifies PD [6]. Similar to AD, treatment of PD aims to address symptoms and slow progression rather than alter the disease course [6].

AD accounts for 60%-80% of all dementia cases, affecting about 24 million people globally. The pathophysiology of AD is characterized by an accumulation of extracellular amyloid plaques and intracellular tau neurofibrillary tangles [6,7]. Research reveals that about 30% of AD cases could be due to modifiable risk factors [8]. Clinically, the disease presents with progressive memory loss and functional loss of independence [6]. Currently, pharmacological treatments can be used to manage symptoms, but do not stop the progression of the disease [9].

ALS, or Lou Gehrig’s Disease, is a degenerative motor neuron disease where the nerve cells and spinal cord are damaged, resulting in muscle weakness and paralysis. ALS has mainly been attributed to gene mutations that cause protein dysfunction, consequently leading to motor defects [6]. Clinical presentation involves focal muscle wasting and atrophy, commonly beginning in the distal muscles [10]. Respiratory failure and death typically occur within three to five years of symptom onset. Contrary to pharmacologic treatment of AD and PD, ALS symptoms are treated with medications that have been shown to delay the progression of the disease [6].

HD is an inherited autosomal dominant neurodegenerative disease. The condition is characterized by a CAG trinucleotide repeat in the Huntingtin (HTT) gene on chromosome 4. Patients with HD typically experience motor symptoms, with chorea being the most specific, as well as cognitive and psychiatric clinical manifestations [11]. Similar to other neurodegenerative diseases, treatment of HD often focuses on managing patients’ symptoms and optimizing quality of life.

Although technology and advances in healthcare have greatly increased the global life expectancy, the new challenges of an aging population are becoming more evident. With age-related conditions such as neurodegenerative diseases becoming more prevalent, research continues to be conducted on non-traditional treatment modalities and modifiable risks of neurodegenerative diseases [12]. The first-line treatments for neurodegenerative diseases involve medications with adverse reactions, making optimizing non-pharmacological modalities essential. Non-pharmacological interventions can include physical therapy, physical activity, and cognitive training, as well as interventions that address diet, obesity, alcohol consumption, and lifestyle choices, all of which may potentially prevent or delay disease onset [13,14]. An additional non-pharmacologic therapy being investigated to manage neurodegenerative diseases is osteopathic manipulative treatment (OMT). Osteopathic medicine is a philosophy and practice based on the premise that the body can inherently heal itself, with one of the roles of the osteopathic physician being to facilitate this ability [15]. One of the central tenets of osteopathic medicine is that the body is a unit, and the person is a unit of body, mind, and spirit. This tenet is crucial to the practice of OMT. OMT is a hands-on treatment modality that addresses a multitude of tissues and bony structures to reintroduce and maintain the body’s natural systemic homeostasis [16].

In contrast to invasive treatments for neurodegenerative diseases, such as deep-brain stimulation, OMT offers a topical, non-intrusive option for adjunctive treatment in patients. There is a plethora of modalities available to osteopathic physicians for the treatment of numerous conditions. These include indirect and direct OMT techniques such as muscle energy technique (MET), high velocity low amplitude (HVLA), myofascial release (MFR), occipito-atlantal decompression (OAD), osteopathic cranial manipulative medicine (OCMM), and balanced ligamentous tension (BLT), among others. MET consists of a patient-produced isometric contraction against a physician-produced counterforce [17]. The most notable findings of MET include an increase in range of motion and lymphatic flow and a decrease in pain, motor excitability, and somatic dysfunction [17,18]. HVLA, a direct technique performed via a rapid, short thrust over a small distance localized to a specific joint, is a rapidly growing OMT modality [16,19]. OCMM is a very broad field consisting of a variety of diagnostic techniques and treatments. OCMM aims to improve brain function and balance cerebrospinal fluid motion [16,20]. MFR uses directional pressure in either an indirect passive manner by following the fascial motion into a position of ease or a direct manner by moving it into a position of resistance [16]. MFR has been shown to release and normalize tissue tone, decrease pain all over the body, and increase range of motion [21,22]. BLT involves placing a joint into a balanced state via compression and movement in different planes to normalize proprioceptive feedback, allowing for ligamentous homeostasis [23]. BLT has been shown to increase the range of motion in structurally diverse joint types, as well as help decrease pain and increase blood and lymphatic flow [24]. In OAD, a non-invasive anterior force is applied to the occipito-atlantal joint, normalizing vagal tone and function through parasympathetic stimulation [25]. The profound effects of this treatment can be seen throughout the body, including but not limited to normalization of cardiac rhythm, anti-inflammatory effects, and increases in intracranial blood flow [25-27].

OMT education provides the osteopathic physician with a vast array of additional treatment options for addressing patient care and improving function and quality of life in a unique and less invasive way. Therefore, the purpose of this study is to map the evidence that pertains to the application of OMT in treating neurodegenerative disorders and identify any gaps that remain in the literature.

## Review

Methods

This scoping review was conducted in accordance with the Joanna Briggs Institute (JBI) methodology for scoping reviews [28] to map the literature regarding the use of OMT in patients with neurodegenerative disorders and identify any gaps in the literature.

Eligibility Criteria

The Population, Concept, Context (PCC) framework was utilized in the development of this study. The population included adult human patients diagnosed with one of the following neurodegenerative diseases: PD, AD, dementia, ALS, and HD. The concept focused on OMT and its potential efficacy and clinical outcomes for these neurodegenerative disorders. The context included all clinical and research settings and all primary research study designs conducted anywhere in the world.

Participants who were not formally diagnosed with these disorders or had a disorder not included in the defined list were excluded. Given the lack of research on human participants regarding OMT for AD, the population criteria were modified to include rats as participants for AD only. Otherwise, the criteria for human participants were strictly maintained for the other four diseases explored. Given that the manipulations used were exclusive to OMT, anything not specified as an OMT technique was eliminated (i.e., physical therapy, chiropractic manipulations, etc.). Any articles that were classified as scoping reviews, meta-analyses, systematic reviews, or editorial papers were excluded, as well as sources not available in full text. Any sources that were translated into English from another language were also excluded. Finally, only studies published after 1995 were included for the purpose of maintaining pertinent and updated information.

Search Strategy

An initial search of the Excerpta Medica database (EMBASE), Ovid Medical Literature Analysis and Retrieval System Online (MEDLINE), and Web of Science was conducted on September 13, 2023. Through the initial search, relevant articles were identified. Searches were conducted independently utilizing the following search terms with abbreviations, truncations, and relevant synonyms added: “osteopathic manipulative treatment” OR “osteopathic manipulation “AND" Alzheimer's disease” OR “neurodegenerative disorders” OR “amyotrophic lateral sclerosis” OR “dementia” OR “Huntington’s disease” OR “Parkinson’s disease”. Additionally, field codes, namely ab (abstracts), ti (titles), and kw (keywords containing OMT and neurodegenerative disorders), were utilized to isolate abstracts, keywords, and titles addressing both OMT and neurodegenerative disorders. This search strategy was adapted for each database. The reference lists for the following articles were screened for additional articles.

Selection of Evidence

Following the search, all identified citations were collected and uploaded into EndNote 21.0.1 / 2023 (Clarivate Analytics, Philadelphia, PA, USA), where any duplicates were removed. Titles and abstracts were screened by independent reviewers for assessment against the inclusion criteria. Potentially relevant sources were retrieved in full text, and their citation details were imported into the JBI System for the Unified Management, Assessment, and Review of Information (JBI SUMARI; JBI, Adelaide, Australia) [29]. The full texts of selected citations were assessed against the inclusion criteria by two independent reviewers. Any disagreements that arose between the reviewers at each stage of the selection process were resolved through an additional reviewer who served as a tiebreaker. The results of the search and the study inclusion process are reported in a Preferred Reporting Items for Systematic Reviews and Meta-Analyses (PRISMA) extension for scoping reviews flow diagram [30].

Critical Appraisal of Sources

A critical appraisal of included articles was performed using the JBI series of checklists that evaluate the quality of a study [31,32]. Two researchers evaluated the included studies using the appropriate JBI appraisal tools. Seven studies were evaluated with the JBI Randomized Controlled Trials checklist, three studies were evaluated with the JBI Quasi-Experimental Studies checklist, and one study was evaluated with the JBI Case Series checklist. Only articles with a JBI appraisal score of 75% or higher, which indicates a low risk of bias, were included in the study.

Data Management and Extraction

The Microsoft Excel software program (Microsoft Corp., Redmond, WA, USA) was used to track and manage collected data. Data was extracted from papers by two or more independent reviewers using a data extraction spreadsheet developed by the reviewers that included the following variables: study author, paper title, study design, origin country of study, study participants, study objective, study methods, and key findings and conclusions. Any disagreements were resolved through a third reviewer who served as a tiebreaker. 

Results

Selected Studies

**Figure 1:**
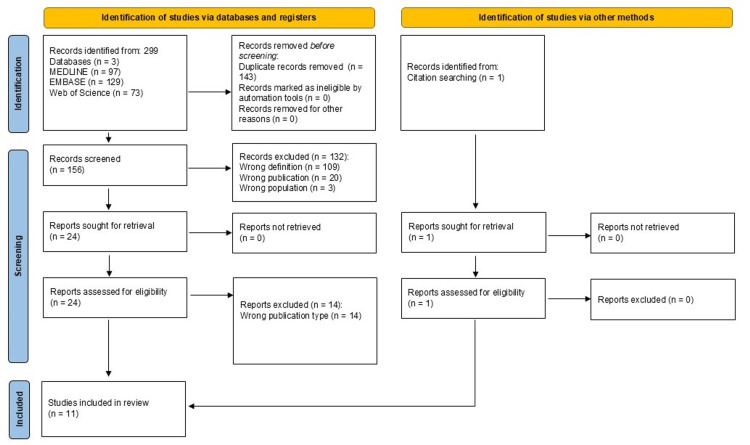
Preferred Reporting Items for Systematic Reviews and Meta-Analyses (PRISMA) flowchart depicting the review process. MEDLINE: Medical Literature Analysis and Retrieval System Online; EMBASE: Excerpta Medica database

Figure 1 presents the PRISMA flowchart depicting the search process. The database and citation searches yielded 300 results, which resulted in 11 articles that met all inclusion criteria.

Of these 11 articles, three studies were found pertaining to AD, one study was found for ALS, and seven studies were found for PD. The studies took place in a total of three countries: the United States (n=8), Italy (n=2), and Germany (n=1). The subjects for the studies included humans (n=8) for the studies pertaining to PD and ALS, as well as rats (n=3) for the studies pertaining to AD. Details regarding the articles are summarized in Table 1.

**Table 1:** Data table showing the data extracted from the 11 papers included in this scoping review Data table showing the data extracted from the eleven papers included in this scoping review. Asterisks (*), when present, signify the number of participants that completed the study from the original pool of participants. PD: Parkinson’s Disease; OMM: Osteopathic Manipulative Medicine; Mini-BESTest: Mini Balance Evaluation Systems Test; SOT: Sensory Organization Test; MDS-UPDRS: Movement Disorder Society Unified Parkinson’s Disease Rating Scale; MDA: Malondialdehyde; DT: Dityrosine; 3-NT: 3-Nitrotyrosine; 8-OHdG: 8-hydroxy-2-deoxyguanosine; OCF: Osteopathy in the Cranial Field; OMT-WB: OMT Whole Body (OMT and OCMM techniques); OMT-ND: OMT Neck Down (only OMY techniques); PD-PS: Parkinson’s Disease with Pisa Syndrome; MIRT: Multidisciplinary Physical Therapy Protocol; ECSA: Eyes Closed Sway Area; EOSA: Eyes Open Sway Area; TLF: Trunk Lateral Flexion; KA: Kyphotic Angle; AD: Alzheimer’s Disease; RCT: Randomized Controlled Trial; OCMM: Osteopathic Cranial Manipulative Medicine; CNS: Central Nervous System; CRI: Cranial Rhythmic Impulse; MWM: Morris Water Maze; IHC: Immunohistochemical; APPsw: Amyloid Precursor Protein; PSI∆E9: Presenilin 1 exon-9 deleted; COM: Cranial Osteopathic Manipulation; ALS: Amyotrophic Lateral Sclerosis; OMT: Osteopathic Manipulative Therapy; SDs: Somatic Dysfunctions; GAS: Goal Attainment Scale; VAS: Visual Analogue Scale; BPI: Brief Pain Inventory short form; McGill QoL: McGill Quality of Life

Author	Title	Design	Origin Country	Sample	Objective	Methods	Results/Conclusion
PD							
DiFrancisco- Donoghue et al., 2017 [33]	Osteopathic Manipulation as a Complementary Approach to Parkinson's Disease: A Controlled Pilot Study	Double-blind RCT	United States	Men and Women diagnosed with PD (n=11*), *9 Men and Women diagnosed with PD (n=11*), *9 completed the study mpleted the study	To ascertain if OMM treatments can improve motor function, postural stability, and balance in individuals with PD.	Participants were randomly assigned to the OMM treatment group or placebo-control counseling group. Each patient's balance was evaluated with Mini-BESTest and SOT, and their overall disease severity with MDS-UPDRS. Patients underwent bi-weekly therapeutic interventions for six weeks, switching groups after six weeks.	No statistically significant changes in SOT or Mini-BESTest in either group (p < 0.05). Statistically significant improvements in UPDRS scores post OMM treatment (p=0.021). Clinically significant improvements in motor function and balance in the OMM treatment group. OMM treatments may be beneficial in improving motor function, no statistically significant changes in SOT or Mini-BESTest in either group (p < 0.05). Statistically significant improvements in UPDRS scores post OMM treatment (p=0.021). Clinically significant improvements in motor function and balance in the OMM treatment group. OMM treatments may be beneficial in improving motor function in patients with PD. on in patients with PD.
Muller and Pietsch, 2013 [34]	Comparison of Gait Training Versus Cranial Osteopathy in Patients with Parkinson’s Disease: A Pilot Study	Double- blind RCT	Germany	Men and Women diagnosed with PD (n= 18*); *17 completed study	To determine the efficacy of gait training and OCF treatments on patients with PD’s gait, as well as compare their effects in a crossover design.	Eligible patients were randomly assigned to the OCF treatment group or the gait training group. Patients’ barefoot walking was analyzed before and after treatment. After one therapeutic intervention, participants switched groups and received another therapeutic intervention.	Statistically significant decrease in walking period for the OCF treatment group (p<0.01)—correlating to increased speed of motion. Statistically significant reduction in the number of steps for the gait training group (p < 0.05). The modalities, gait training, and OCF treatments may improve different aspects of gait in patients with PD.
Terrell et al., 2022 [35]	Effects of Osteopathic Manipulative Treatment vs. Osteopathic Cranial Manipulative Medicine on Parkinsonian Gait	RCT	United States	Men and women diagnosed with PD (n=45*) and healthy age-matched controls (n=45**). 41 PD participants completed the study.*43 healthy controls completed the study.	To evaluate the effects of a singular OCMM treatment and/or OMT on patients with PD's gaits.	Participants were randomly assigned to the OMT-WB group, the OMT-ND group, or the sham group. Gait information was collected before and after a single treatment session via a standardized treadmill motion capture system. Gait and ROM of the hip, knee, and ankle were measured before and after the treatment session.	Statistically significant increase in sagittal hip range of motion in the OMT-WB group (p=0.038). There was no statistically significant difference in sagittal hip, knee, or ankle angles via waveform analysis between any of the groups. OMT, along with OCMM, could be beneficial in improving gait in individuals with PD.
Wells et al., 1999 [36]	Standard Osteopathic Manipulative Treatment Acutely Improves Gait Performance in Patients with Parkinson's Disease.	QED	United States	Men and women diagnosed with PD with gait impairment (n= 20) and healthy control volunteers (n=8)	To determine the effects of a singular OMT session on patients with PD's gait. To display how quantitative gait analysis may be effectively used for examining the outcomes of OMT on patients with PD.	Participants with PD were assigned to either the OMT group or a sham treatment group. All controls were in the OMT group. Every participant underwent gait analysis (evaluating stride length, cadence, and velocities) before and after a single treatment.	Participants with PD demonstrated a statistically significant increase in stride length (p< 0.02) and cadence (p< 0.005) compared with their pre-OMT values. Control participants in the OMT group and sham-treated PD participants did not show statistically significant changes in gait post-treatment compared with their pretreatment values. OMT may be able to provide significant increases in patients with PD’s gaits in a single session.
Zarucchi et al., 2020 [37]	Efficacy of Osteopathic Manipulative Treatment on Postural Control in Parkinsonian Patients with Pisa Syndrome: A Pilot Randomized Placebo-Controlled Trial	Single-blind RCT	Italy	Men and women diagnosed with PD-related Pisa syndrome (N= 24)	To evaluate the efficacy of OMT on postural control in PD-PS who are undergoing a multidisciplinary intensive rehabilitation treatment protocol.	Eligible participants with PD-PS were randomly assigned to the OMT group (OMT + MIRT) or the sham-OMT (sham + MIRT) group. Participants’ ECSA, EOSA, TLF, and KA were assessed before and after treatments. Outcome assessment is blind to participants’ group allocation. Four treatments over 30 days in conjunction with standard physical therapy protocol.	Participants with PD-PS in the OMT group demonstrated statistically significant decreased sway area (p= 0.01) and TLF inclination reduction of 3.33 degrees (p= 0.044) in comparison to the placebo group. For patients with PD-PS, OMT in conjunction with MIRT may provide benefits in postural control and improvements in TLF.
Docherty et al., 2022 [38]	Preliminary Effects of Osteopathic Manipulative Medicine on Reactive Oxygen Species in Parkinson's Disease: A Randomized Controlled Pilot Study	Non-blinded RCT	United States	Men and women diagnosed with PD (n= 32*); *19 completed study	To investigate the effects of OMT on oxidative stress biomarkers in PD.	Participants were randomly assigned to either the counseling group or the OMT group. Biomarkers of oxidative stress (MDA, DT, 3-NT, 8-OHdG, 8-isoprostan e) were measured before and after the first session and at weeks 3, 6, and 10.	No statistically significant changes in oxidative stress marker levels were found in blood plasma during intervention or after compared to controls (p > 0.05). OMT did not influence the preselected oxidative stress biomarkers.
Mancini et al., 2021 [39]	Gut Microbiome Changes with Osteopathic Treatment of Constipation in Parkinson's Disease: A Pilot Study	Case Series	United States	Men and women diagnosed with PD with chronic constipation (n= 9*). *6 completed the study.	To evaluate the impact of OMM treatments on chronic constipation and ascertain if OMM treatments can alter the gut microbiome in patients with PD.	Participants’ constipation severities were evaluated, and stool samples were collected before and after OMT. Stool samples were evaluated via the Bristol Stool scale and microbiota analysis with a diversity score and 16S ribosomal RNA sequencing. Participants underwent four weeks of weekly OMT intervention.	Statistically significant decrease in mean constipation severity (p=0.010) and increase in mean quality of life (p=0.041) post OMM. Bristol stool scales improved from type 2 to type 3 post-OMM treatments but did not reach statistical significance (p = 0.167). Statistically significant change in gut phyla post-OMM. OMM treatments may be beneficial in treating constipation and altering the gut microbiome in patients with PD.
AD dementia							
Anandakrishnan et al., 2022 [40]	Cranial Manipulation Affects Cholinergic Pathway Gene Expression in Aged Rats	QED	United States	Aged rats (n=12*); used six rats (three treatment, three control) as their RNA sequences were of high enough quality to be evaluated.	To examine OCMM’s effects on gene expression in the context of improving spatial memory.	Rats were assigned to the treatment group or the control group. Rats in the treatment group underwent OCMM (CV4 technique) daily for seven days. Rats’ RNA samples were extracted and sequenced.	Increased expression of 36 genes involved in neuronal pathways in treated rats (FDR < 0.004) compared to the control group. Of note, the genes Slc5a7, Chat, Slc18a3, Adcy5, and Cacna2d2, involved in acetylcholine neurotransmission, had the largest changes from the control group to the treatment group. OCMM treatments may provide a low-risk, beneficial treatment of age-related dementia and AD.
Tobey et al., 2019 [41]	Effect of Osteopathic Cranial Manipulative Medicine on an Aged Rat Model of Alzheimer's Disease	RCT	United States	Aged male rats (n=12)	To evaluate whether OCMM influences spatial memory and affects CNS waste clearance.	Rats were randomly assigned to either an untreated or an OCMM treatment group (using the CV-4 technique once daily for seven days). MWM assays were performed pre-/post-treatment on rats injected with florbetapir F18 radiotracer, which binds Aβ plaques. PET & CT imaging and subsequent IHC & Western blot analysis were performed.	The OCMM treatment group displayed significant improvement in spatial memory after seven days in comparison to the untreated group (p < 0.01). The OCMM treatment group’s hemispheres demonstrated reduced beta amyloid levels (p < 0.01), reduced activated astrocytes (p < 0.001), and improved neurotransmission (p < 0.001) in comparison to the untreated group. OCMM may affect molecular mechanisms related to AD.
Tobey et al., 2020 [42]	Mechanoceutics Alters Alzheimer's Disease Phenotypes in Transgenic Rats: A Pilot Study	QED	United States	TgF344-AD Rats (n = 9) with K595N and M596L gene mutations affecting APPsw and PSI∆E9 cause early-onset familial AD.	To identify the effects of COM on AD behavioral and biochemical processes.	Rats were assigned to the COM treatment group or the untreated group. The COM group received COM daily for seven days. MWM assays were performed pre-/post-treatment. Rats were injected with the florbetapir F18 radiotracer and underwent PET imaging.	The COM treatment group displayed enhanced MWM assay performance in comparison to the untreated group (p < 0.05). The COM treatment group demonstrated reduced Aβ signals (p < 0.01) and Aβ peptide (p < 0.05). Western blots revealed that activation of astrocytes in the treatment group was significant in comparison to the untreated group (p < 0.05). COM treatments may mediate changes in behavioral and biochemical processes in the context of AD.
ALS							
Maggiani et al., 2016 [43]	Osteopathic Manual Treatment for Amyotrophic Lateral Sclerosis: A Feasibility Pilot Study	Single- blind RCT	Italy	Men and women diagnosed with ALS (n=14) and healthy age-matched controls (n=14)	To evaluate the practicality, safety, and satisfaction of OMT in ALS patients.	Participants with ALS were randomly assigned to the OMT group or the standard care group. Blind osteopathic assessment of SDs for GAS calculation. OMT group: treatment weekly for four weeks and fortnightly for eight weeks. Standard care group twice-weekly physiotherapy. OMT open period weekly treatment for participants with ALS (n=10) for eight weeks. Participants were evaluated with a BPI and McGill QoL. VAS was used for the OMT group.	Participants displayed high satisfaction with OMT. OMT is indicated to be safe and feasible. No statistically significant differences were seen in GAS between groups.

PD

The effects of OMT on various aspects of PD were studied. Five studies focused on the interaction between osteopathic manipulative medicine (OMM) and walking mechanics, gait, postural stability, or balance in patients with PD [33-37]. Other studies evaluated the relationship between OMM and oxidative stress markers [38] or normalized gut microbial abundance [39].

Studies that assessed PD involved an amalgam of patients diagnosed with PD and healthy controls. Subjects included patients diagnosed with PD in general [33-36, 38], patients with PD chronic constipation [39], or patients with PD-associated Pisa Syndrome [37]. Studies that used control groups were distinguished by either using a non-physical contact group, such as a counseling treatment [33, 38], or a physical contact group, “sham-OMT” [36, 35]. One study randomized subjects into “whole body” OMT groups (OMT-WB), including OCMM, or neck-down OMT (OMT-ND) [35]. Other studies compared OMT to alternative therapies using a physiotherapy gait training treatment group [34] or an OMT and multidisciplinary physical therapy protocol (MIRT) treatment group [37].

Following biweekly OMM treatments for six weeks, participants with PD showed statistically significant improvements in their Movement Disorder Society-Unified Parkinson’s Disease Rating Scale (MDS-UPDRS) score compared to the PD participants in the control group [33]. The PD participants did not show statistically significant improvements in their Sensory Organization Test (SOT) score or their Mini-Balance Evaluation Systems Test (mini-BESTest) scores when compared to the PD participants in the control group; however, there was improvement noted for SOT and mini-BESTest in both the OMT treatment group and control group [33].

After two treatments, walking evaluations revealed that participants with OMT-treated PD displayed a decrease in period and thus an increase in motion implementation but not step frequency compared to the participants with PD treated with physiotherapy [34]. Following a single OMT-WB treatment, participants with PD demonstrated a statistically significant increase in their hip range of motion, while OMT-ND was not as effective [35]. There was no statistically significant improvement in knee or ankle range of motion or difference in hip, knee, or ankle joint waveforms for participants with PD treated with OMT-WB, OMT-ND, or sham-OMT [35]. In response to a single OMT treatment session, participants with PD demonstrated a significant increase in gait parameters related to stride and velocity for the upper and lower limbs compared to their pretreatment values [36].

Pisa syndrome is a PD-associated postural abnormality characterized by lateral flexion of the trunk. This posture is most prominent while the patient is standing or walking. Participants with PD-associated Pisa syndrome treated with four sessions of OMT plus MIRT displayed a statistically significant decrease in eyes-closed sway area (ECSA) compared to the sham-OMT plus MIRT group [37]. There was not a statistically significant difference in change in trunk lateral flexion (TLF) or kyphotic angle (KA) between the two treatment groups; however, the relationship between the OMT plus MIRT treatment regimen and mean TLF angle reduction, as well as reduction in KA, was statistically significant [37]. There was also a statistically significant association between the change in ECSA and the change in TLF observed in the OMT plus MIRT treatment group [37].

In regard to PD-related conditions, following twice-weekly OMT sessions for six weeks, participants with PD did not show statistically significant changes in their selected blood plasma and urinary oxidative stress markers (MDA, Dt, 3-NT, 8-OHdG, 8-isoprostane) when compared to the control treatment group [38]. In response to weekly OMM treatments for four weeks, the participants with PD-associated chronic constipation demonstrated statistically significant improvements in Patient Assessment of Constipation Symptoms (PAC-SYM), Patient Assessment of Quality of Life (PAC-QOL), and Wexner Cleveland Constipation Scoring System (WCCSS) compared to their pre-OMM ratings [39]. The participants with PD chronic constipation had statistically significant decreases in members of the phylum Firmicutes and statistically significant changes in the abundance of members of the phylum Actinobacteria in their stool samples following their OMT sessions [39]. This did not result in statistically significant improvements in their Bristol stool scale compared to their pre-OMM ratings; however, the rating improved clinically from type 2 to type 3 [39].

AD Dementia

Three rat studies, but no human studies, were found regarding AD and OMT. These rat studies evaluated OMT’s impact, especially that of OCMM, on AD dementia in a preclinical rat model [40-42]. To evaluate the effect of OCMM on AD-related gene expression, seven days of daily OCMM treatment were performed. 426 genes in the treatment group were found to be statistically significantly differentially expressed (SDE) post-treatment (FDR < 0.004), with 314 genes that were overexpressed and 112 genes that were underexpressed [40]. Thirty-six of these SDE genes were involved in the neuronal pathway. The top five SDE genes with the largest fold change were part of the cholinergic neurotransmission mechanism, known to affect cognitive function. Additionally, 39.9% of the SDE genes have been previously implicated in neurological disorders [40].

Spatial memory changes in rat subjects after OCMM treatment were evaluated in two studies [41, 42]. The subjects included aged rats [41] or rats with gene mutations linked to early-onset familial AD [42]. Following OCMM treatment, there was statistically significant improved spatial memory (p< 0.01) [41], reduced amyloid β levels (p< 0.01) [41,42], activated astrocytes (p< 0.001) [41] (p< 0.05) [42], improved neurotransmission (p< 0.001) [41], and improved performance (p< 0.05) [42] in comparison to the control rat subjects. The visual cortex, retrosplenial cortex, and pituitary gland were the three areas in rats where there was the greatest numerical reduction in signal [42]. There were statistically increased cytokine levels of RANTES, EOTAXIN, IL-1, IL-10, IL-17A (p<0.05), and IL-13 (p<0.01) biomolecules in rats, indicating an immune response to OCMM [42].

ALS

One study evaluated OMT’s effects on patients diagnosed with ALS [43]. It was noted at the start of the study that the ALS participants randomly assigned to the OMT group had significantly faster Disease Progression Index (DPI), suggesting more advanced diseases and shorter disease durations than the ALS participants in the standard of care group. The study’s participants reported high satisfaction following OMT. Results revealed no statistically significant decreases in pain modification evaluated with the Brief Pain Inventory or increases in quality of life evaluated with the McGill quality of life assessment following treatment or between the OMT group and standard of care group [43].

HD

Although HD was included in the scoping review search criteria, no articles were found linking HD with OMT treatment modalities.

Discussion

Eleven articles were identified from existing literature regarding OMT and its use for treating neurodegenerative disorders, namely PD, AD, and ALS, and no articles were identified on HD. Though the studies yielded promising results for the role of OMT as an adjunct therapy in the management of neurodegenerative diseases, the number of articles illustrates the paucity of research that has been conducted. 

PD

The seven articles focused on motor and postural impacts of OCMM and OMT on PD patients and produced statistically significant data in support of improved gait kinematics and posture from treatment. Since parkinsonian gait and postural changes are risk factors for falls, the results display the potential of osteopathic treatment as an adjunct therapy, alongside traditional physiotherapy and medication, in improving the quality of life for affected patients. Improvements in total hip range of motion [35], stride length and cadence [34,36], ECSA, TLF [37], and UPDRS scores [33] can help PD patients maintain their mobility and avoid additional risks associated with falls. These motor benefits can also give patients, and by extension their loved ones, peace of mind regarding physical safety.

Furthermore, chronic constipation is one of the early signs of PD that commonly manifests prior to motor symptoms. One of the identified studies explores the possible link between changes in gut microbiota that occur secondary to OMM-based treatment in PD patients. Researchers performed different OMM treatment methods to normalize autonomic nervous system (ANS) dysregulation, improve pelvic floor muscle tone, increase gastrointestinal motility, and release myofascial restrictions within the diaphragm. They observed significant improvements in microbiota diversity after four weekly OMM treatments; this included a significant shift in the balance of phyla of bacteria, improvement in mean constipation severity, and improvement in quality of life scores. Researchers found that the microbiota that increased post OMM treatment have been strongly correlated with mobility, gait, and posture; however, they believed the techniques were only indirectly the cause of the change in microbiota [39]. Though the mechanism for this observation is still hypothetical, by increasing movement through the gastrointestinal system, new bacteria may have been introduced and developed residence after moving residual stool through the body. This may have improved the patients’ overall microbial counts and diversity, leading to decreased constipation and increased quality of life.

Neurodegeneration is commonly linked with processes such as neuroinflammation and with reactive oxygen species (ROS). There were no significant changes in blood plasma levels of the ROS markers MDA, DT, 3-NT, or 8-OHdG after OMM [38]. Although ROS levels did not change, subjects and caregivers reported increased stability and ease of movement after completing this study [38]. This suggests that while OMT may help with increasing the movement and physical stability of patients, it may not alter biomarkers.

Limitations to these studies on the benefits of OMT and OCMM for PD patients include small sample sizes and a lack of longitudinal follow-up. Two studies were also limited by the inconsistent conditions between the control group and the OMT-allocated cohort, as the control groups had no physical contact with the physician or OMT practitioner [33, 38]. This lack of therapeutic touch in the control groups could have affected the results of the studies, as the act of a physician simply laying hands on a patient, even without osteopathic treatment, can impact that patient’s condition. Terrell et al. also point out that a lack of statistically significant changes in certain kinematic metrics could be due to the stage of disease not being controlled for [35]. While studies can certainly have too narrow a focus, it may be beneficial to understand the motor benefits of OMT at each stage of disease symptomatology. As a disease progresses, the management must evolve with it.

Finally, three studies’ designs incorporated a 12-hour washout period from the participants’ last PD medication administration before taking measurements [33, 38, 36]. Given that OMT is an adjunct therapy, it stands to reason that most PD patients who present for OMT will also be taking medications such as carbidopa-levodopa. Therefore, it is essential to understand how OMT will work to improve patients’ symptoms in combination with medications and any other therapies they might be receiving. Future studies on OMT’s effects on PD should be implemented to better understand the benefits of this treatment. Studies should additionally elaborate on whether OMT can be used as a stand-alone treatment in certain instances or if other treatments, such as medications, should be continued in conjunction with OMT.

AD Dementia

All three AD dementia articles generated by our search utilized rats as test subjects and exclusively utilized OCMM techniques, primarily compression of the fourth ventricle (CV4) manipulation [40-42]. OCMM was found to alter cholinergic neuronal genes that have been linked to cognitive function in a manner consistent with better memory [40]. Researchers postulated that gene expression could be altered by affecting intracranial pressure, inducing cellular stress, and increasing lymphatic venous return [40]. OCMM was also found to improve spatial learning and memory, reduce amyloid β protein levels, alter amyloid β levels associated with upregulated lymphatic vessels, and modulate synaptic transmission [41, 42].

The CV4 technique in particular involves mechanical pressure applied to the occiput, medial to the connection of the occiput and temporal bone, and inferior to the lambdoid suture. This pressure aids in increasing flow throughout the brain, leading researchers to believe that improved fluid circulation from the CV4 technique results in improved metabolic waste clearance throughout the brain. Overall, CV4 was shown to be an effective treatment for rat models with AD [40-42]. Finally, OCMM increased cytokine levels of IL-1, IL-10, IL-13, RANTES, IL-17A, and EOTAXIN. Through this data, it was suggested that the immune system was able to recognize pressure applied to the cranial region during treatment with CV4 and alter important functions within the body [42].

Although these research studies made significant progress in identifying the positive benefits of OCMM as a treatment for AD dementia, there were major limitations. All three of these studies included only rat models as subjects, with a limited sample size. Future studies should focus on human participants versus control subjects to further evaluate if OCMM may benefit individuals living with AD dementia.

ALS

In a single study, OMT was deemed feasible and safe for ALS patients regardless of the stage of disease [43]. ALS patients who were assigned OMT reported high levels of satisfaction with the treatment, with no adverse effects noted. Patients allocated to the OMT group reported a higher rate of goal attainment, though the sample size was too small to reach significance [43].

The study was further limited by the tendency of OMT-allocated subjects to have a faster-progressing variant of ALS. As that illness encompasses a spectrum of diseases that progress at variable rates, it is difficult for a small sample size comprising primarily rapidly progressing variants to provide a detailed picture of what benefits OMT offers ALS patients. Furthermore, the disconnect between patient-reported satisfaction with OMT and the lack of change in QoL or pain may indicate that the standardized questionnaires covering those concerns are not well-suited for ALS patients’ experiences [43]. Further research to better ascertain the benefits of OMT in the management of ALS should include larger cohorts of subjects covering early-to-late-stage disease and should be performed with surveys whose questions are specifically written for ALS symptoms and complications.

HD

No articles were found linking HD with OMT treatment modalities. It should be noted that there are many variations in terminology surrounding OMT and OCMM, and that it is therefore possible that such articles do exist and that they were not captured by our search methodology or were unavailable in the databases we searched. However, the availability of such studies for other neurodegenerative diseases such as PD, AD, and ALS suggests that there is a dearth of research into the safety, feasibility, and possible benefits of OMT for HD patients. Given the similarities between symptoms seen in HD and other neurodegenerative diseases, it should be possible to design a pilot study into a preliminary OMT protocol. These studies could begin with small cohorts before increasing sample size as safety and feasibility are established.

Scoping Review Limitations

In addition to the individual limitations of the studies outlined above, there are limitations associated with the performance of this scoping review. The limited number of databases utilized may have missed some articles on the subject. The search terms used may not have included every potential spelling, synonym, or variation of each term. Finally, excluding articles that were not written in English may have introduced a language bias that reduced the potential number of search results returned. 

## Conclusions

OMT shows potential as an adjunct treatment modality for those suffering from neurodegenerative disorders. Improved posture, stability, and gait were all observed in PD patients after treatment with a wide range of OMM techniques. Although ROS levels in PD patients did not improve after OMT, patients had a significant positive impact on microbiota diversity and reported quality of life. Among rat models with AD, OCMM improved spatial learning and memory, reduced amyloid β protein levels, and modulated synaptic transmission. OMT was also found to alter cholinergic gene expression and amyloid β levels associated with upregulated lymphatic vessels, potentially indicating a mechanism to combat the dementia associated with AD. Though these results from rats are promising for adjunctive treatments for AD, all currently identified literature is preclinical in nature and represents a gap in the literature. The implementation of OMT in the management of ALS symptoms was determined to be safe and practical, though further studies, better tailored to the unique experience of ALS patients, are warranted to fully understand the treatments’ benefits. Finally, although HD was included in the database search, no articles could be found specifically mentioning OMT treatments used for these patients. More research studies need to be conducted to determine the full benefits of OMT in patients with neurodegenerative disorders. The OMT treatments explored in future research should be varied in methodology and patient-centered, consistent with osteopathic principles. In addition to increasing awareness of OMT through education, there should also be greater advocacy for its integration into clinical practice.
